# The Relationship between Whole-Milk Dairy Foods and Metabolic Health Highlights an Opportunity for Dietary Fat Recommendations to Evolve with the State of the Science

**DOI:** 10.3390/nu15163570

**Published:** 2023-08-13

**Authors:** Moises Torres-Gonzalez

**Affiliations:** National Dairy Council, 10255 West Higgins Road, Suite 900, Rosemont, IL 60018, USA; moises.torres-gonzalez@dairy.org

**Keywords:** dietary fat, obesity, whole-milk dairy foods, saturated fat, metabolic disorders, dietary fat guidance

## Abstract

The science of dietary fats has evolved, and a body of evidence indicates they are complex bioactive nutrients that have different effects on health depending on their food source, chain length, degree of saturation, and other factors that can be affected by food processing, handling, and storage. As such, it is becoming increasingly clear that the effects of foods on obesity and metabolic health cannot be predicted simply with their fat content. The aim of this opinion article is to provide a brief overview of select recent research on the effects of whole-milk dairy foods on body composition and indicators of metabolic health across the lifespan to show the gap between current knowledge and dietary guidance. As the state of the science on dietary fats and human health evolves to consider the complexity of food matrices, the total nutrient package they deliver, and the health impacts associated with dietary patterns, so too must guidelines for dietary fat.

## 1. Dietary Recommendations Evolve with the State of Nutrition Science

In this opinion piece, a brief overview is provided of select recent research on the effects on whole-milk dairy foods on body composition and indicators of metabolic health across the life span to support the hypothesis that a gap exists between current knowledge and dietary guidance. For over a century, nutrition science has driven dietary guidance, with the earliest objectives being to ensure nutrient adequacy to prevent diseases of deficiency [1]. Recent emphasis has shifted, however, to mitigating consequences associated with excess energy, such as obesity, type 2 diabetes, and cardiovascular disease (CVD) [2]. More than half a century ago, dietary fat became a target, and up until 2002, dietary guidance in the United States focused on limiting total fat consumption to less than 30% of calories with the idea that low-fat diets would help prevent obesity and CVD [1]. Clinical and observational research indicated otherwise, demonstrating no strong health advantages to lowering total dietary fat in foods or dietary patterns [3]. Guidelines have since evolved to include a more moderate range of dietary fat intake of 20–35% of calories [4]. Current recommendations emphasize replacing saturated fat with dietary mono- and polyunsaturated vegetable oils to reduce the risk of CVD and help maintain a healthy weight [4]. The American Heart Association presidential advisory on dietary fats and CVD found, in a review of randomized controlled trials, that lowering saturated fat intake using replacement with polyunsaturated vegetable oil reduced CVD by approximately 30%—a reduction similar to that of treatment with statins [5]. Furthermore, saturated fat reduction was also associated with lowered blood LDL cholesterol, a validated risk factor for coronary artery disease that links clinical evidence with prospective observations [5]. There is strong evidence to support the benefits of consuming PUFA, omega-6 PUFA, and seafood-derived omega-3 PUFA for cardiometabolic health [3]. 

Nutrition science has continued to evolve, and now recognizes that the impact of diet cannot be extrapolated from any single nutrient or surrogate outcome marker [3]. Dietary fats are complex nutrients that not only provide a concentrated source of energy but are also an integral part of cellular structure and function as well as biochemical and physiological pathways critical to metabolism [6,7]. The health impact of these nutrients cannot be categorized simply by their degree of saturation [8,9,10]. For instance, the “Western” dietary pattern has been associated with an increased ratio of omega-6 to omega-3 fatty acids from 1:1 to 20:1, which has been associated with obesity and detrimental effects on human health [11]. As such, whereas current evidence strongly supports cardiometabolic benefits from the consumption of polyunsaturated fats, it also indicates that considering food sources of dietary fat may be most meaningful when linking diet and health, particularly because saturated, monounsaturated, and polyunsaturated fats are present in a variety of complex food matrices that have been associated with cardiometabolic benefits [3]. In this regard, a prospective cohort analysis of 120,877 males and females in the United States enrolled in The Nurses’ Health Study (NHS), the NHS II, and the Health Professionals Follow-up Study showed that specific foods were independently associated with long-term weight gain [12]. For example, weight gain after 4 years was most associated with the consumption of potato chips, potatoes, sugar-sweetened beverages, and unprocessed and processed meats. Weight loss after 4 years was most associated with the consumption of vegetables, whole grains, fruits, nuts, and yogurt. There were no significant differences detected in weight associated with the consumption of composite whole-fat dairy foods, whole-fat milk, cheese, composite low-fat dairy foods, and low-fat or skim milk after 4 years of follow up. Other lifestyle factors associated with weight change included physical activity, alcohol use, smoking, sleep, and television watching. The results of this large prospective analysis demonstrated independent relationships between specific foods or beverages and long-term weight gain that were not predictable based on total and saturated fat content [12]. Weight, however, may not be the best predictor of metabolic health. More recently, the sagittal abdominal diameter (SAD), a predictor of abdominal fat, was utilized in a cross-sectional analysis of data from 13,544 U.S. adults from the 2011–2016 NHANES to evaluate the relationship between milk-fat consumption and obesity. A significantly lower SAD, which has been demonstrated to be a better predictor of cardiometabolic health than weight and BMI, was associated with the regular consumption of fat-free milk, despite a significantly lower BMI being associated with both consistent fat-free and whole-milk consumption [13]. Additionally, the 2020–2025 Dietary Guidelines for Americans emphasize that dietary patterns may better predict overall health and disease risk than individual foods or nutrients [4].

## 2. Whole-Milk Dairy Foods Are Just One Example of the Complexity of Food Sources of Fat and Their Effects on Body Composition and Metabolic Health

Dairy foods comprise complex matrices that deliver nutrients and bioactive ingredients with different effects on long-term weight control and metabolic health [14,15]. Comparatively, whole- and reduced-fat dairy foods contain more calories and saturated fat than low-fat and fat-free versions, but a growing body of evidence from observational and intervention studies indicates that the association between dairy food consumption and body weight cannot be predicted based solely on calorie and fat content [16]. A meta-analysis of thirteen cohort studies on coronary heart disease (CHD) and seven on ischemic stroke indicated that total, low-fat, and high-fat dairy consumption had no association with CHD or stroke, respectively [17]. Another meta-analysis of twenty-nine cohort studies reported no associations between total dairy consumption, including high- and low-fat varieties, and CHD or CVD [18]. Likewise, a meta-analysis of thirteen studies found no associations between total dairy consumption and risk for CHD, regardless of fat content [19]. In another meta-analysis of thirteen cohort studies, whereas total dairy consumption was not associated with CVD risk in males, it was inversely related to CVD risk in females [20]. The results indicated a potential benefit from including whole-milk dairy consumption in female diets. A criticism of these studies is that they are based on a self-reported intake of dairy foods, which is subject to recall bias and under-reporting.

There is also robust clinical evidence to support observations that dairy consumption, regardless of fat content, does not increase the risk for CVD. A trial that aimed to test a higher-fat version of the Dietary Approaches to Stop Hypertension (DASH) demonstrated that the inclusion of whole-milk dairy foods improved triglycerides and had no significant effects on other risk factors for CVD compared with the standard DASH diet, despite a significant increase in saturated fat consumption among healthy middle-aged males and females [21]. Another clinical trial involving 111 adults with type 2 diabetes demonstrated no statistical differences in the glucose control, body weight, BMI, waist circumference, or waist-to-hip ratio between participants instructed to consume three or more servings of low-fat and fat-free dairy or whole-milk dairy daily for 24 weeks [22]. More recently, a randomized controlled trial that aimed to compare the effects of diets rich in low-fat or full-fat dairy foods, including milk, cheese, and yogurt, on glucose tolerance in adults, ranging from 18–75 years of age, with metabolic syndrome found no intervention effect on glucose tolerance [23]. Researchers reported that both the low-fat and high-fat dairy diets significantly decreased the Matsuda insulin sensitivity index, a whole-body insulin sensitivity score that is based on a composite estimate of hepatic and muscle insulin sensitivity [23]. In a crossover intervention conducted in males and females with elevated blood pressure, the addition of whole milk or whole-milk dairy foods to a diet without dairy for 4 weeks resulted in no significant differences in vascular function between groups [24]. Another randomized controlled trial involving sixty-six adults with metabolic syndrome demonstrated no significant differences between consuming 3.3 servings daily of either low-fat or whole milk, cheese, and yogurt for 12 weeks on fasting serum total LDL or HDL cholesterol, triglycerides, free fatty acids, or blood pressure [25]. 

Similar results have been reported in children and adolescents. A systematic review of observational and intervention studies assessing associations between whole- and reduced-fat dairy intake and measures of adiposity and biomarkers of cardiometabolic disease risk in children and adolescents 2–18 years of age found that cross-sectional, longitudinal, and intervention studies were consistent in finding that whole-milk dairy foods were not associated with increased measures of weight gain or adiposity [16]. Similar findings were reported from two recent prospective cohort analyses. One prospective analysis of 7467 children, aged 9 months to 8 years, found that whole-milk consumption was associated with 16% lower odds of an overweight status and 18% lower odds of obesity, after an average 2.7-year follow up [26]. Another prospective study of 796 children, aged 3 years, reported that the early childhood consumption of higher-fat compared with lower-fat milk was associated with lower odds of an overweight status or obesity in early adolescence [27]. Further, whole-milk dairy foods were not associated with increased cardiometabolic risk, except in one double-blind controlled trial that found a change from whole to reduced-fat milk consumption significantly decreased LDL-C, apoA-1, and apoB in adolescents [16]. A systematic review and meta-analysis of three prospective cohort studies and eleven cross-sectional studies that evaluated the relationship between whole and reduced-fat milk consumption and adiposity in children and adolescents 1–18 years of age found that the odds of an overweight status or obesity were 49% lower among those who consumed whole compared with reduced-fat milk (95% CI: 0.52, 0.72; *p* < 0.0001) [28]; however, the heterogeneity of studies was high (I2 = 73.8%) and warrants further investigation [28]. A randomized controlled trial conducted on adolescent females to determine whether increased dairy food consumption as part of a lifestyle intervention promoted favorable changes to body composition demonstrated that whereas weight did not significantly change in any group, the group consuming four servings of dairy foods per day, including low-fat milk, low or reduced-fat Greek yogurt, and 42 g of cheddar or marble full-fat cheese, featured a significantly decreased fat mass and increased lean mass more than the low-dairy and control groups [29]. The study demonstrated that saturated fat contained in whole-milk dairy foods improved body composition in the absence of weight loss [29].

A review of the mechanisms by which the dairy matrix impacts human health highlighted that the inclusion of dairy foods, including milk, cheese, and yogurt of any fat variety, may beneficially impact cardiometabolic health via beneficial effects on inflammatory pathways in the gastrointestinal tract, liver, and vasculature [30]. Taken together, findings from a body of literature on whole-milk dairy food consumption and cardiometabolic health complicate the simple notion that foods containing fat induce obesity and cardiometabolic risk based on their total fat, saturated fat, and calorie contents [15]. 

## 3. There Is an Opportunity for Dietary Fat Recommendations to Have a Greater Impact on Reducing the Risk of Obesity and Its Health Consequences

Dietary guidance from authoritative sources has the potential to affect the health of billions of people worldwide. Thus, it is of the utmost importance that guidelines represent the current state of nutrition science. At present, many dietary guidelines around the world recommend reduced-fat diets to help mitigate unhealthy weight gain in adults without differentiating between food sources of dietary fats or even the diversity of fatty acids within different classes of fats. The World Health Organization (WHO) suggested that nutrition transition—a shift from traditional diets composed of whole foods to diets composed of processed foods that are energy-dense and nutrient-poor—was an important factor associated with the increased incidence of an overweight status and obesity in low- and middle-income countries [31]. This is of importance because the metabolic diseases that were once associated with the “Western diet” of higher-income countries are now on the rise in lower-income countries, with 82% of premature deaths being from non-communicable diseases occurring in low- and middle-income countries [32]. Rather than emphasizing what has already been learned from large cohorts in high-income countries that are already featuring chronic metabolic disease and that nutrition strategies focused on single nutrient reductions are outdated, future recommendations should focus on the consumption of nutrient-rich, whole-food dietary patterns that have been demonstrated to improve cardiometabolic health [4]. Unfortunately, the WHO proposed recommendations based on dated views of dietary fat, such as, “To avoid unhealthy weight gain, total fat should not exceed 30% of total energy intake [31].” Dietary patterns that include whole foods such as fruits, vegetables, eggs, cheese, milk, meat, and fish, regardless of fat content, could improve the nutrition transition in some low- and middle-income countries by promoting overall healthy dietary patterns, and thus having a positive impact on cardiometabolic health.

## 4. Conclusions

Dietary recommendations focused on limiting individual nutrients have not successfully reduced the incidence of obesity and chronic diseases globally. Reductionist dietary fat recommendations that consider fat only as a concentrated fuel source or ignore the complexity of fatty acids, the whole-food matrices, and the broader nutrient package from which they are derived do not represent the current state of the science on dietary fats and human health [15]. There is an opportunity for dietary fat guidance to consider the full breadth of evidence available, and to consider the evolution of dietary recommendations by health professionals who recognize the importance of whole foods and dietary patterns over single, isolated nutrients. Recommendations for dietary fat consumption could be carried out while simultaneously considering the food source, presence of other nutrients, food matrices, and health impacts associated with dietary patterns. Ultimately, the goal should be to align policy with sound science that leads to improved diet quality and long-term health.

## Data Availability

Not Applicable.
